# Prenatal PFAS exposure and offspring health: evidence review and implications for intergenerational risk assessment

**DOI:** 10.3389/fpubh.2026.1892134

**Published:** 2026-07-16

**Authors:** Bin Deng, Xuan Xia, Xiaoxiang Sun

**Affiliations:** Department of Pharmacy, Union Hospital, Tongji Medical College, Huazhong University of Science and Technology, Wuhan, China

**Keywords:** epigenetic programming, offspring health, per- and polyfluoroalkyl substances, placental transfer, prenatal exposure

## Abstract

Prenatal exposure to per- and polyfluoroalkyl substances (PFAS) represents a critical developmental concern because several PFAS cross the placenta and may perturb biological programming during sensitive windows. Although epidemiological and experimental studies have linked prenatal exposure to several measured PFAS, primarily legacy perfluoroalkyl acids such as perfluorooctanoic acid (PFOA), perfluorooctanesulfonic acid (PFOS), Perfluorohexane Sulfonic Acid (PFHxS), and Perfluorononanoic Acid (PFNA), to diverse offspring outcomes, the strength of evidence differs substantially across compounds and health domains. Current human evidence is strongest for impaired vaccine antibody responses and altered growth or metabolic trajectories in studies dominated by legacy PFAS, whereas evidence for other congeners, short-chain PFAS, ether-based alternatives, and fluorotelomer compounds remains sparse. Mechanistically, placental transfer, nuclear receptor perturbation, thyroid hormone transport disruption, mitochondrial stress, immune modulation, and epigenetic reprogramming may jointly contribute to developmental susceptibility. We further discuss how emerging PFAS alternatives and real-world mixture exposures challenge single-chemical and adult-centered assessment paradigms. Finally, we outline a conceptual framework for developmental hazard assessment, in which exposure-window characterization, congener-specific toxicokinetics, human-relevant models, multi-omics biomarkers, and PBPK/PBTK modeling are positioned as complementary components rather than as a fully operational assessment system.

## Graphical Abstract

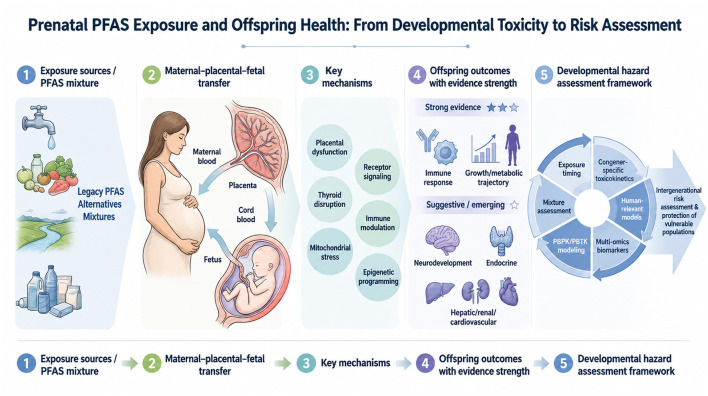

## Highlights

• Prenatal exposure to legacy PFAS, especially PFOA, PFOS, PFHxS, and PFNA, is linked to impaired vaccine responses and altered offspring growth.
• Evidence for neurodevelopmental, endocrine, hepatic, renal, and cardiovascular effects remains limited, particularly for emerging PFAS.
• Placental transfer, endocrine disruption, immune modulation, and epigenetic changes may drive developmental toxicity.
• PFAS alternatives and mixture exposures challenge conventional single-chemical risk assessment.

- A developmental hazard framework integrating exposure timing, toxicokinetics, biomarkers, and PBPK/PBTK modeling is proposed.

## 1 Introduction

Per- and polyfluoroalkyl substances (PFAS) comprise a large class of synthetic organofluorine chemicals widely used in industrial processes and consumer products because of their oil- and water-repellent properties (1). Their highly stable carbon-fluorine bonds confer environmental persistence, long-range transport potential, and, for several congeners, prolonged biological half-lives (2). Despite often being treated as a single group, PFAS comprise thousands of structurally diverse compounds with varying degradation potentials. Table 1 summarizes their classification and representative examples (3). The phase-out of legacy PFAS such as perfluorooctanoic acid (PFOA) and perfluorooctanesulfonic acid (PFOS) has led to the increasing use of alternative compounds such as 6:2 chlorinated polyfluoroalkyl ether sulfonate (6:2 Cl-PFESA), whose environmental behavior and health risks remain insufficiently understood, raising emerging concerns (4).

**Table 1:** Key categories of PFAS: properties and exemplary compounds.

Category	Definition and examples	Key characteristics	Toxicokinetic and regulatory notes
Legacy long-chain PFAAs	**Carbon chain length ≥ 6 (PFSAs), or ≥ 7 (PFCAs)** PFOA, PFOS, PFNA, PFDA, PFUnDA, PFDoDA	•High persistence and bioaccumulation potential •Strong protein binding in serum (primarily albumin) •Long elimination half-lives in humans •High octanol-water partition coefficients •Resistant to hydrolysis, photolysis, and biodegradatior	•Primary targets of global regulation (Stockholm Convention, EPA PFOA Stewardship Program) •Extensive human biomonitoring data available •Established adverse health outcomes: thyroid disease, elevated cholesterol, immunotoxicity •Phase-out initiated in early 2000s
Short-chain PFAAs	**Carbon chain length < 6 (PFSAs), or < 7 (PFCAs)** PFBA, PFBS, PFPeA, PFHxA, PFHpA	•High water solubility and environmental mobility •Lower bioaccumulation potential compared to long-chain analogs •Still extremely persistent (resistant to degradation) •Shorter biological half-lives in organisms •Greater tendency to partition to water phase	•Common replacements for long-chain PFAAs in industrial applications •Challenge for drinking water remediation due to high mobility •Increasing detection frequency in groundwater and surface water •Toxicological data gaps remain; emerging evidence of similar health effects
PFAA precursors	**FTOHs:** 4:2 FTOH, 6:2 FTOH, 8:2 FTOH, 10:2 FTOH **FOSA/FOSEs:** N-EtFOSA, N-EtFOSE, N-MeFOSA, N-MeFOSE **FTSAs:** 6:2 FTSA, 8:2 FTSA	•Can degrade/transform to form terminal PFAAs through atmospheric oxidation or metabolic biotransformation •Complex exposure assessment due to multiple transformation pathways •Volatile precursors (FTOHs) contribute to long-range atmospheric transport •Biotransformation rates vary by structure and environmental conditions	•Indirect and prolonged source of PFAAs in environment and biota •Important for indoor air and dust exposure (especially FTOHs from consumer products) •Often overlooked in conventional PFAS monitoring programs •Precursor biotransformation complicates source apportionment
Emerging alternatives	**GenX Chemicals:** HFPO-DA, HFPO-TA, ADONA **Chlorinated Polyfluoroalkyl Ether Sulfonates:** 6:2 Cl-PFESA, 8:2 Cl-PFESA **Other Novel Structures:** PFMPA, PFECHS	•Designed as “safer” alternatives with improved degradability claims •Emerging environmental concerns due to widespread detection •Distinct toxic modes of action suspected (different receptor binding, metabolic pathways) •Structural modifications alter physicochemical properties	•Limited toxicity and monitoring data available •Already detected in environment (water, sediment, biota) and humans (serum, breast milk) •Subject of intense ongoing research and regulatory scrutiny •Regulatory frameworks lagging behind commercial introductior •Potential for regrettable substitution scenarios

PFAS, per- and polyfluoroalkyl substances; PFAAs, perfluoroalkyl acids; PFSAs, perfluoroalkyl sulfonic acids; PFCAs, perfluoroalkyl carboxylic acids; PFOA, perfluorooctanoic acid; PFOS, perfluorooctane sulfonic acid; PFNA, perfluorononanoic acid; PFDA, perfluorodecanoic acid; PFUnDA, perfluoroundecanoic acid; PFDoDA, perfluorododecanoic acid; PFSA, perfluoroalkane sulfonic acids; PFBA, perfluorobutanoic acid; PFBS, perfluorobutanesulfonic acid; PFPeA, perfluoropentanoic acid; PFHxA, perfluorohexanoic acid; PFHpA, perfluoroheptanoic acid; FTOHs, fluorotelomer alcohols; FOSA/FOSEs, perfluoroalkyl sulfonamides; GenX, hexafluoropropylene oxide dimer acid; HFPO-DA, hexafluoropropylene Oxide Dimer Acid; 6:2 Cl-PFESA, 6:2 chlorinated polyfluoroalkyl ether sulfonate.

**Figure 1:**
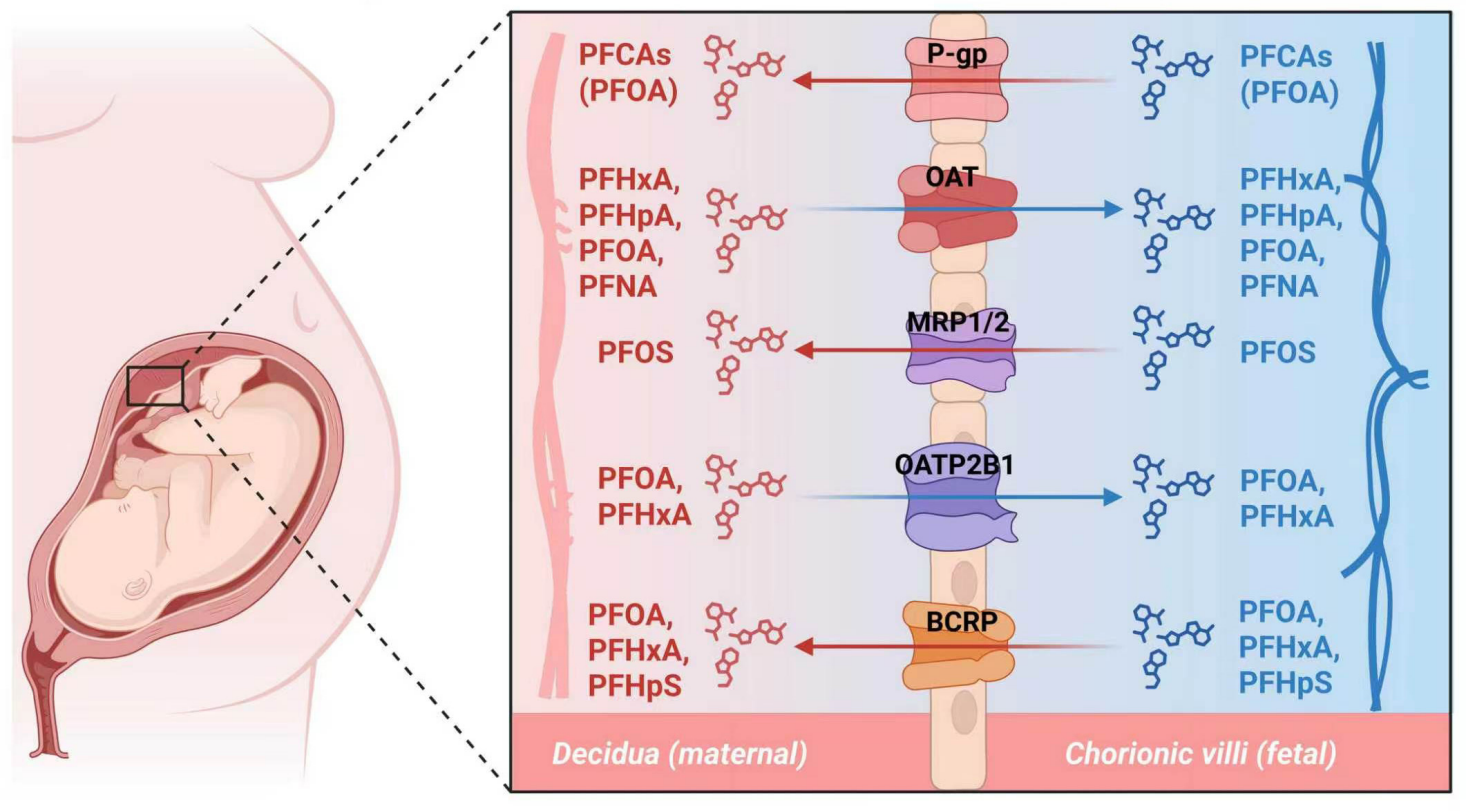
Transporters in the Placenta Involved in PFAS Transport. PFAS, per- and polyfluoroalkyl substances; PFCA, perfluoroalkyl carboxylic acid; PFOA, perfluorooctanoic acid; PFHxA, perfluorohexanoic acid; PFHpA, perfluoroheptanoic acid; PFOA, perfluorooctanoic acid; PFNA, perfluorononanoic acid; PFOS, perfluorooctane sulfonic acid; PFHpS, perfluoroheptane sulfonic acid; P-gp, P-glycoprotein; OAT, organic anion transporter; MRP, multidrug resistance-associated protein; OATP2B1, organic anion transporting polypeptide 2B1; BCRP, breast cancer resistance protein.

Pregnancy represents a uniquely vulnerable window for PFAS toxicity (5). Multiple PFAS can cross the placental barrier, as demonstrated by their detection in umbilical cord blood, placental tissues, and fetal organs, confirming direct in utero exposure (6). Notably, placental transfer efficiency varies across PFAS compounds depending on carbon chain length and functional groups, likely mediated by differential interactions with placental transporters, as schematically summarized in Figure 1 (7, 8). Postnatally, breastfeeding represents an additional and sustained exposure pathway (9). Elevated PFAS levels in breast milk have been reported in certain regions, with estimated infant intakes potentially exceeding health-based guidance values. Such exposure has been associated with impaired growth and increased obesity risk, particularly in female offspring (10, 11). Collectively, prenatal and early-life exposure constitutes a sensitive window for PFAS-related intergenerational effects, driven by immature physiological systems and limited metabolic clearance capacity (12).

The Developmental Origins of Health and Disease (DOHaD) framework provides a biologically plausible basis for linking prenatal PFAS exposure to later disease susceptibility. It posits that the intrauterine environment shapes lifelong physiological function, and that exposures during critical developmental windows can reprogram biological systems, increasing susceptibility to metabolic and other chronic diseases later in life (13, 14). Accumulating evidence indicates that PFAS act as environmental stressors that disrupt developmental programming through multiple mechanisms, including receptor-mediated signaling [e.g., peroxisome proliferator-activated receptors (15), thyroid hormone receptors (16)], induction of oxidative stress (17), and epigenetic modifications [e.g., DNA methylation (18, 19) and histone alterations (20)]. These changes can impair metabolic, neurological, and immune systems (21). Thus, within the DOHaD framework, prenatal PFAS exposure can be viewed not merely as a toxicological event, but as an early-life determinant of disease risk, highlighting the importance of protecting vulnerable developmental windows to reduce the burden of non-communicable diseases.

Because PFAS comprise a chemically diverse class, evidence should not be interpreted as uniformly applicable to all congeners. In this review, the term “PFAS” is used as an umbrella term only when referring to the chemical class or mixed exposure scenarios. When discussing health-effect evidence, “PFAS” generally refers to the compounds measured in the cited studies, which are predominantly legacy perfluoroalkyl acids, especially perfluorooctanoic acid (PFOA), perfluorooctanesulfonic acid (PFOS), Perfluorohexane Sulfonic Acid (PFHxS), and Perfluorononanoic Acid (PFNA). Evidence for short-chain PFAS, ether-based substitutes, fluorotelomer compounds, and other emerging alternatives remains limited and is discussed separately where available. Therefore, conclusions are framed as congener-specific or evidence-domain-specific rather than as class-wide toxicity claims.

Several authoritative agencies have conducted comprehensive PFAS assessments, including European Food Safety Authority (EFSA), United States Environmental Protection Agency (US EPA), and International Agency for Research on Cancer (IARC) (22–25). These assessments provide essential regulatory and hazard-evaluation foundations, but their primary objectives differ from those of the present review. EFSA derived a group tolerable intake for selected PFAS, mainly based on immune effects (22, 25); US EPA developed chemical-specific toxicity values for PFOA and PFOS to support drinking-water regulation (23); and IARC evaluated the carcinogenicity of PFOA and PFOS (24). These assessments are therefore indispensable but are not specifically designed to synthesize prenatal exposure windows, placental transfer, fetal and infant toxicokinetics, offspring health trajectories, developmental mechanisms, multi-omics biomarkers, and intergenerational risk-assessment implications in a single developmental framework. The present review is positioned as a complementary developmental-health synthesis rather than a replacement for regulatory hazard assessments.

Therefore, the unique contribution of this review is not to duplicate existing regulatory hazard assessments, but to reorganize the PFAS evidence through a developmental and intergenerational lens. Specifically, this review integrates prenatal exposure timing, placental transfer, fetal and infant toxicokinetics, offspring health trajectories, mechanistic biomarkers, emerging PFAS alternatives, mixture exposure, and Physiologically Based Pharmacokinetic (PBPK)/ Physiologically Based Toxicokinetic (PBTK)-based internal dose reconstruction into a single conceptual framework for developmental hazard assessment.

This review therefore aims to provide an evidence-graded and mechanism-informed synthesis of prenatal PFAS exposure and offspring health. Specially, we: (i) summarize epidemiological evidence across immune, metabolic, neurodevelopmental, endocrine, and emerging health outcomes; (ii) distinguish stronger evidence from suggestive or limited evidence; (iii) integrate placental transfer, receptor-mediated pathways, mitochondrial and inflammatory stress, immune modulation, and epigenetic programming into a developmental mechanism framework; (iv) discuss emerging alternatives and mixture exposures as major challenges for causal inference and risk assessment; and (v) outline a conceptual developmental hazard assessment framework and clarify how exposure timing, PFAS-specific toxicokinetics, mechanistic biomarkers, human-relevant models, and (PBPK)/(PBTK) modeling could be integrated in future studies.

## 2 Epidemiological landscape: linking prenatal PFAS to adverse offspring outcomes

This section provides a descriptive overview of epidemiological findings across major offspring health domains. This review synthesizes epidemiological evidence linking prenatal or early-life PFAS exposure with offspring health outcomes, emphasizing prospective cohorts, longitudinal studies, systematic reviews, and meta-analyses. As a narrative, evidence-graded synthesis rather than a formal systematic review, we searched PubMed/MEDLINE, Web of Science, Scopus, and Embase up to 2026 using PFAS, exposure-window, and offspring-outcome terms; reference lists of relevant reviews and meta-analyses were also screened.

Eligible studies assessed prenatal or early-life PFAS exposure in relation to offspring immune, growth/metabolic, neurodevelopmental, endocrine, hepatic, renal, or cardiovascular outcomes. Experimental studies were included when they provided mechanistic support. Evidence quality was assessed using domains adapted from the Newcastle–Ottawa Scale and environmental-health frameworks, including study design, temporality, exposure and outcome assessment, confounding control, sample size, attrition, statistical methods, and reporting transparency. Two reviewers independently screened and appraised the included studies. Disagreements were resolved through discussion, and when consensus could not be reached, a third reviewer adjudicated. Because this article is a narrative evidence review rather than a full systematic review, we did not apply Grading of Recommendations Assessment, Development and Evaluation (GRADE) as a formal quantitative grading system. Instead, we used a predefined qualitative grading framework adapted from the Newcastle–Ottawa Scale, the Navigation Guide, the Office of Health Assessment and Translation (OHAT) risk-of-bias principles, and Bradford Hill considerations. To improve reproducibility, qualitative descriptors used in the evidence-grading framework were operationalized before outcome-level grading. For epidemiological evidence, a small cohort was defined as fewer than 500 mother–child pairs or outcome-specific participants, a moderate cohort as 500–1,999 participants, and a large cohort as 2,000 or more participants. “Limited risk of bias” indicated that most key domains were adequately addressed, including prospective exposure assessment, valid outcome measurement, adjustment for major confounders, acceptable attrition, and transparent reporting. “Moderate risk of bias” indicated concerns in one or two key domains that were unlikely to fully explain the observed association. “Serious risk of bias” indicated major concerns in multiple domains, such as cross-sectional design, substantial exposure misclassification, inadequate confounding control, high or differential attrition, or selective reporting. These thresholds were used to support transparent and reproducible qualitative grading across outcome domains. Table 2 illustrates the criteria for grading the level of evidence Outcome-specific evidence was graded as strong, suggestive, or limited based on consistency, study quality, biological plausibility, and risk of bias. To further strengthen causal interpretation, we also used a Bradford Hill-informed qualitative appraisal, considering temporality, consistency, strength of association, dose–response evidence where available, biological plausibility, coherence with mechanistic evidence, and alternative explanations such as residual confounding and exposure misclassification. Because dose–response patterns were not assessed uniformly across studies, we did not assume a single universal dose–response shape. Instead, we considered whether available studies supported monotonic, threshold-like, or potentially non-monotonic associations for each outcome domain where sufficient evidence was available. Table 3 summarizes the classified evidence.

**Table 2:** Criteria for outcome-level evidence grading.

Grade	Criteria
Strong evidence	Supported by ≥3 independent prospective cohorts or at leastone high-quality meta-analysis/systematic review; includes at leastone large cohort (≥2,000 participants) or multiple moderate cohorts (500–1,999 participants); findings are generally consistent in direction; temporality is clear; exposure and outcome assessment are valid; major confounders are adjusted; risk of bias is limited; and biological plausibility is supported by mechanistic evidence.
Suggestive evidence	Supported by ≥2 independent studies, usually including at leastone prospective cohort; sample sizes are small to moderate (< 2,000 participants in most studies); findings are generally similar in direction but not fully consistent; some concerns exist regarding exposure timing, outcome heterogeneity, confounding adjustment, or replication; risk of bias is moderate; and biological plausibility is present but not definitive.
Limited evidence	Supported by onlyone study, small cohorts (< 500 participants), cross-sectional or retrospective designs, inconsistent findings, limited replication, serious concerns about exposure or outcome assessment, inadequate confounding control, high attrition, or weak mechanistic support. Evidence is insufficient for firm causal interpretation.

**Table 3:** Strength of evidence for associations between prenatal pfas exposure and offspring health outcomes.

Health outcome domain	Specific outcome	Strength of evidence	Key supporting findings	Main PFAS congeners supporting the evidence	Notable effect modifiers	Bradford Hill-informed appraisal	Reference
**Immune function**	Reduced vaccine antibody response	**Strong**	• Consistent inverse associations across multiple birth cohorts • Dose-response reported for tetanus, diphtheria, and rubella antibodies • Associations remain after confounder adjustment and are mechanistically plausible	Mostly PFOA, PFOS; some PFHxS, PFNA	• Often stronger in females • Modified by PFAS mixture composition and timing of pregnancy exposure	Stronger support: temporality, consistency, plausibility, coherence	(37, 38)
	Increased respiratory infections	**Strong/Suggestive**	• Higher risks of bronchiolitis, bronchitis, pneumonia, and recurrent infections in early childhood • Observed mainly in prospective cohorts with prenatal exposure assessment • Likely related to impaired immune competence	PFOA, PFOS, PFHxS, PFNA; inconsistent by congener	• Limited modifier data • Possible interaction with environmental tobacco smoke		
**Metabolic health**	Altered birth weighte	**Strong**	• Meta-analyses show a robust inverse association with birth weight • Findings are consistent across populations and regions • Effect sizes are modest but statistically significant	PFOA, PFOS, PFHxS, PFNA	• Potential interaction with maternal pre-pregnancy BMI • Few consistent modifiers identified	Stronger support: temporality, consistency, plausibilty, population relevance	(26)
	Increased childhood BMI/obesity	**Strong**	• Associated with accelerated postnatal weight gain and higher BMI z-scores • Links persist after controlling for birth weight and maternal factors • Supports metabolic programming and adipogenesis pathways	Mainly PFOA, PFOS, PFHxS	• Often stronger in females • Typically emerges at 5–8 years and varies with postnatal growth pattern		
	Early biomarkers of NAFLD	**Suggestive**	• Associated with elevated ALT/GGT, liver stiffness, and steatosis indices in childhood • Evidence supports early hepatic effects but clinical progression data are limited	Mainly PFOA, PFOS, PFHxS	• Potential interaction with childhood obesity • Modifier data remain sparse		
**Neurodevelopment**	Reduced cognitive function	**Suggestive**	•Lower intelligence, working memory, and executive function scores reported •Observed across several birth cohorts for both individual PFAS and mixtures •Effect sizes are modest but meaningful at the population level	PFOA, PFOS, PFHxS, PFNA; limited data for alternatives	•Possible interaction with maternal thyroid status •No consistent modifier pattern established	Suggestive: temporality, consistency, plausibility, population relevance	(27, 28)
	ADHD-related behaviors	**Suggestive**	•Linked to ADHD diagnosis and higher symptom scores for inattention and hyperactivity •Supported by both parent- and teacher-reported outcomes	PFOA, PFOS, PFHxS, PFNA; inconsistent by congener	•Often stronger in males •May be modified by genetic susceptibility and prenatal stress		
**Endocrine function**	Altered thyroid hormone levels	**Suggestive**	•Associated with altered cord blood TSH/FT4 and maternal thyroid profiles during pregnancy •Patterns vary by PFAS compound but support thyroid-axis disruption	PFOA, PFOS, PFHxS, PFNA; limited data for alternatives	•Possible interaction with maternal iodine status •Modifier data are limited	Suggestive: plausibility present, heterogeneity and replication limitations	(45, 46)
	Altered pubertal timing	**Limited/Suggestive**	•Earlier menarche in girls and delayed pubertal development in boys have been reported •Evidence remains limited by few longitudinal studies with repeated pubertal assessment	Sparse; mostly legacy PFAS, few studies on alternatives	•Strong sex-specific pattern •May interact with childhood adiposity and co-exposure to other endocrine disruptors		

Evidence grades were assigned at the outcome-domain level using the predefined criteria shown in Table 2. Grades reflect qualitative confidence in the body of evidence rather than formal GRADE certainty ratings. PFAS, per- and polyfluoroalkyl substances; PFCA, perfluoroalkyl carboxylic acid; PFOA, perfluorooctanoic acid; PFHxA, perfluorohexanoic acid; PFHpA, perfluoroheptanoic acid; PFOA, perfluorooctanoic acid; PFNA, perfluorononanoic acid; PFOS, perfluorooctane sulfonic acid; PFHpS, perfluoroheptane sulfonic acid; BMI, body mass index; NAFLD, non-alcoholic fatty liver disease; ALT, alanine aminotransferase; GGT, gamma-glutamyl transferase; ADHD, attention-deficit/hyperactivity disorder; TSH, thyroid-stimulating hormone; FT4, free thyroxine; WISC, Wechsler intelligence scale for children; WPPSI, Wechsler preschool and primary scale of intelligence; HOME, health outcomes and measures of the environment; DRD4, dopamine receptor D4; DAT1, dopamine transporter 1.

### 2.1 Impaired growth and metabolic dysregulation

Prenatal PFAS exposure influences offspring growth and metabolism in a stage-dependent manner. Epidemiological studies consistently associate prenatal PFAS exposure with an increased risk of small for gestational age (SGA), indicating impaired fetal growth (26). This pattern often shifts postnatally, with some cohorts reporting accelerated BMI gain and adiposity in early life, with more pronounced associations in girls in several studies (27, 28). These altered growth trajectories are accompanied by early metabolic disturbances. Prenatal PFAS exposure has been associated with biomarkers of non-alcoholic fatty liver disease in childhood (29), including elevated alanine aminotransferase (ALT) and gamma-glutamyl transferase (GGT) levels (30), as well as increased adiposity, particularly central fat accumulation, as demonstrated by imaging-based studies such as dual-energy X-ray absorptiometry (DXA) (31). In parallel, disruptions in glucose and lipid metabolism have been reported in exposed offspring (32). Overall, current evidence suggests a transition from fetal growth restriction to postnatal metabolic dysregulation, which may increase the risk of metabolic syndrome later in life.

### 2.2 Neurodevelopmental toxicity and cognitive deficits

Prenatal PFAS exposure has been associated with adverse neuropsychological outcomes in offspring. Birth cohort studies and pooled analyses link prenatal exposure to impaired cognitive function in childhood, including reduced intelligence and deficits in learning and memory (33). Prenatal PFAS exposure has also been associated with increased risk of attention deficit/hyperactivity disorder (ADHD), with some studies reporting stronger associations in boys (34). In addition, PFAS exposure has been linked to delays in social communication and persistent behavioral problems from infancy to school age, particularly in emotional regulation and executive function (35, 36). Overall, current evidence suggests that prenatal PFAS exposure may broadly disrupt cognitive, behavioral, and social development.

### 2.3 Immune system dysfunction and increased susceptibility

Prenatal PFAS exposure elicits immunotoxic effects on offspring. Epidemiological studies consistently associate in utero PFAS exposure with suppressed vaccine antibody responses, particularly against tetanus and diphtheria, with effects most evident in infancy and early childhood (37, 38). Prospective cohort studies further link prenatal PFAS exposure to increased susceptibility to infectious diseases, including more frequent respiratory infections and higher risks of bronchitis and pneumonia (39, 40). prenatal PFAS exposure has been associated with increased risks of asthma and allergic diseases, including atopic dermatitis and allergic rhinitis, although sex-specific patterns for allergic and respiratory outcomes remain less consistently replicated (41, 42). Overall, current evidence indicates that PFAS can disrupt immune development and increase the risk of both infectious and allergic diseases in offspring.

### 2.4 Endocrine disruption: thyroid and reproductive hormones

As established endocrine-disrupting chemicals, several measured legacy PFAS have been associated with altered endocrine markers through disruption of the thyroid and gonadal axes (43, 44). Birth cohort studies have consistently associated prenatal PFAS exposure with altered neonatal thyroid function, particularly elevated thyroid-stimulating hormone (TSH) and reduced free thyroxine (FT4) levels, suggesting disruption of fetal thyroid development after placental transfer (45, 46). Prenatal PFAS exposure has also been linked to altered reproductive development, including earlier menarche in girls and delayed genital development in boys, including sex-specific effects on pubertal timing (47, 48). Moreover, PFAS exposure has been associated with altered cord blood sex hormone levels, with legacy PFAS such as PFOS and PFOA linked to reduced testosterone in male infants (49). Collectively, these findings suggest that PFAS disrupt multiple hormonal pathways during development, with potential long-term consequences for endocrine health.

### 2.5 Other emerging health endpoints

Beyond the major systems discussed above, emerging evidence suggests that prenatal PFAS exposure may also affect hepatic, cardiovascular, and renal function in offspring. In children, cord blood PFAS levels have been associated with disrupted lipid metabolism and increased susceptibility to liver injury (50, 51). Birth cohort studies further report associations between prenatal PFAS exposure and elevated blood pressure in childhood and adolescence, with some studies suggesting stronger associations in boys, although replication remains limited (52, 53). In addition, prenatal PFAS exposure has been linked to higher urinary biomarkers of renal tubular damage in newborns (54). Epigenetic studies demonstrate an inverse association between prenatal PFAS exposure and telomere length in umbilical cord blood, pointing to a potential role in accelerated cellular aging (55).

## 3 Unraveling the mechanisms: from placental transfer to developmental programming

**Figure 2:**
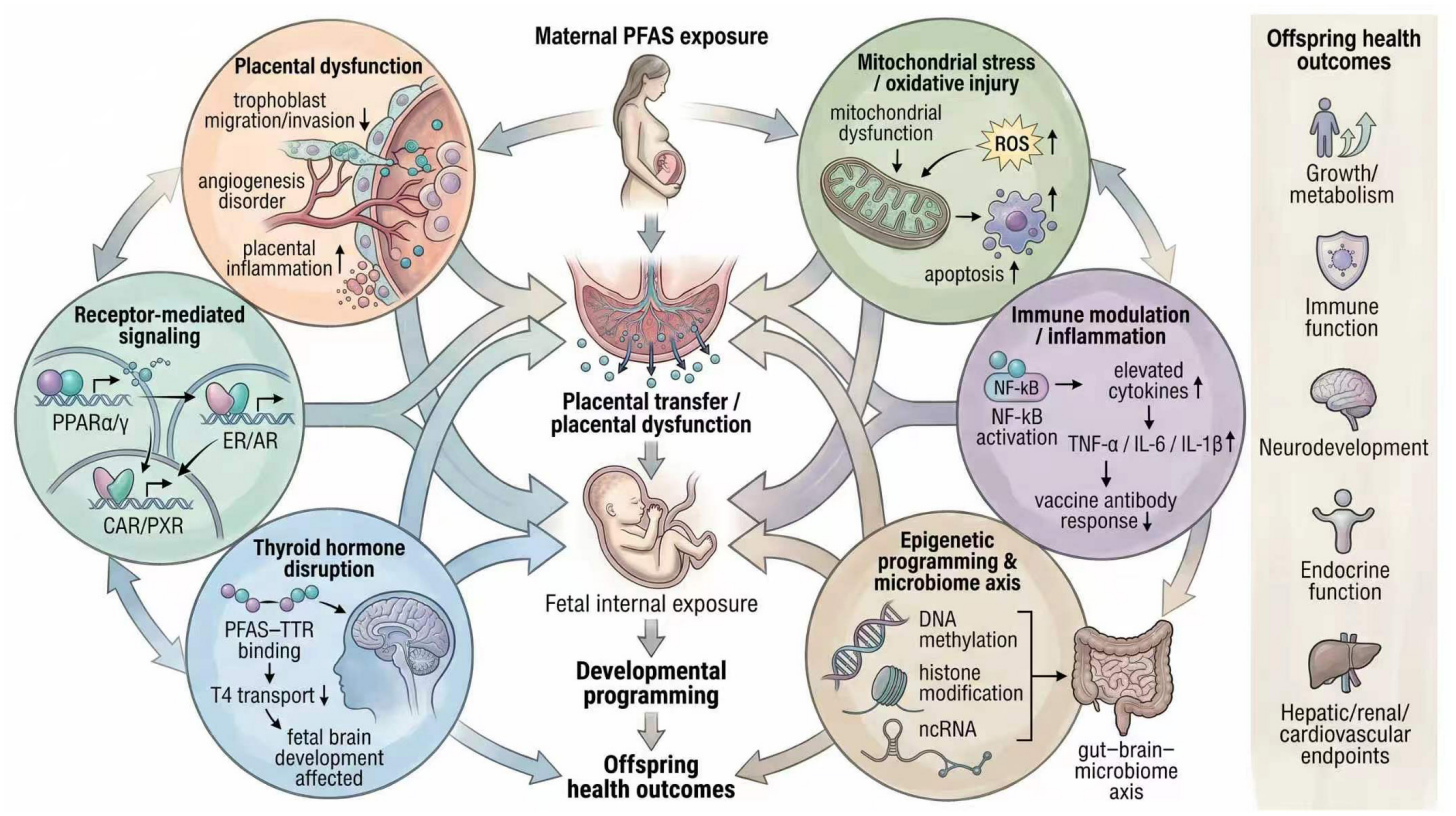
Mechanistic pathways linking prenatal PFAS exposure to offspring developmental toxicity. Key mechanisms of pathophysiological changes in offspring caused by PFAS exposure during pregnancy. PFAS, per- and polyfluoroalkyl substances; PPAR, peroxisome proliferator-activated receptor; TTR, thyroid hormone transport; ER, estrogen receptor; AR, androgen receptor; CAR, constitutive androstane receptor; PXR, pregnane X receptor; TNF-α, tumor necrosis factor-alpha; IL, interleukin.

Mechanistic evidence is uneven across PFAS congeners and should be interpreted according to biological level and pathway specificity. Most receptor-binding, oxidative-stress, endocrine-disruption, and epigenetic studies have focused on PFOA, PFOS, PFHxS, PFNA, or selected emerging substitutes such as hexafluoropropylene oxide dimer acid (GenX) and 6:2 Cl-PFESA. Therefore, the mechanisms summarized below are organized hierarchically from the maternal–fetal interface and placental function, to molecular initiating events, cellular stress responses, epigenetic regulation, and microbiome-related pathways. These mechanisms should be viewed as congener-specific or pathway-specific evidence rather than uniform mechanisms shared by all PFAS. Figure 2 provides an overview of the proposed mechanistic pathways.

**Figure 3:**
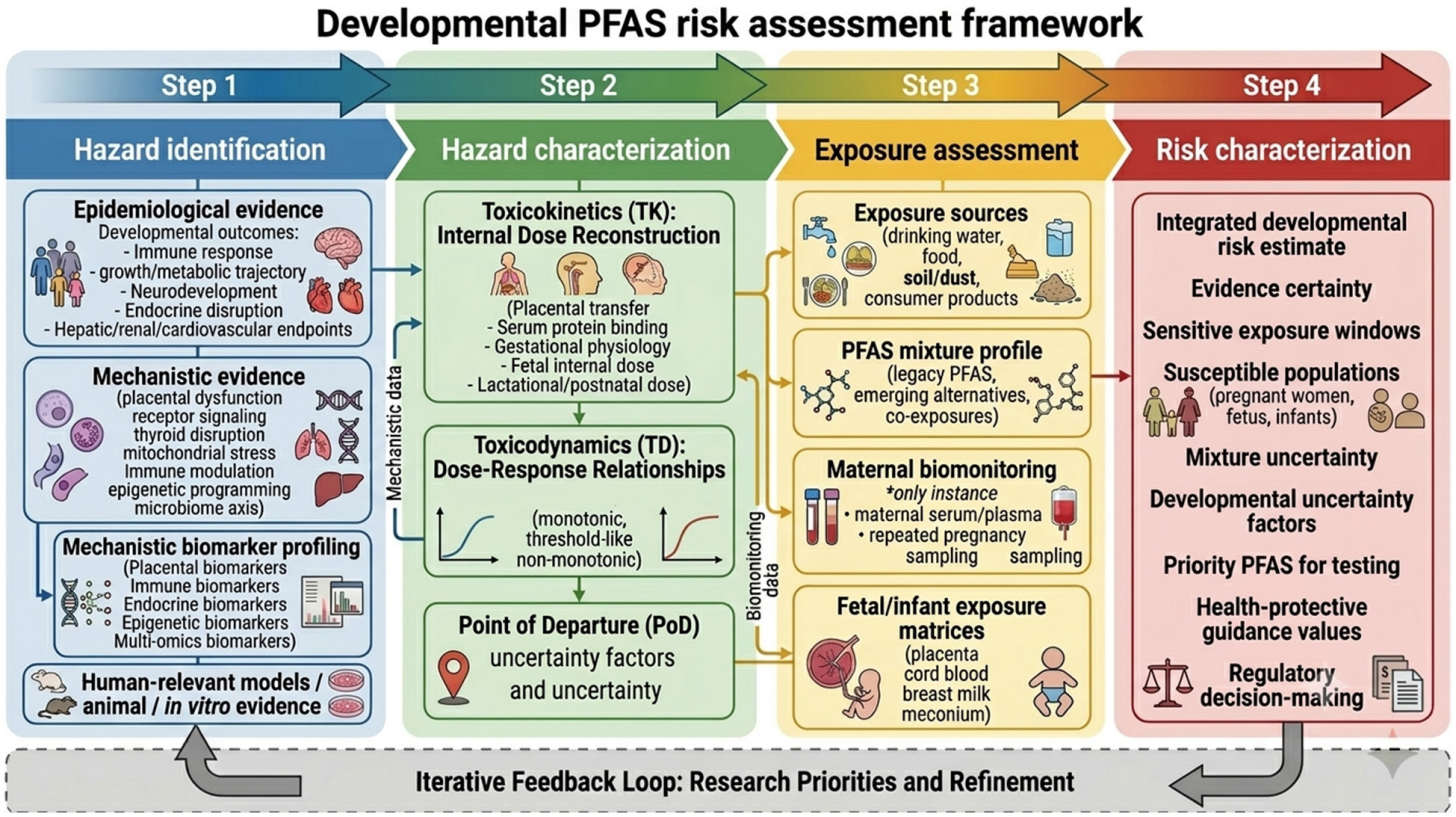
Developmental PFAS risk assessment framework based on the internationally recognized four-step paradigm. The revised framework follows the sequential order of hazard identification, hazard characterization, exposure assessment, and risk characterization. Dose–response assessment and derivation of health-based guidance values, such as TDI, ADI, and RfD, are positioned under hazard characterization. Exposure assessment is placed as the third step and includes external exposure sources, food and water intake data, PFAS concentrations in relevant media, biomonitoring matrices, exposure timing, and mixture exposure. Risk characterization integrates hazard characterization and exposure assessment to estimate the probability and severity of adverse developmental effects in exposed populations. PFAS-specific toxicokinetic features, including long biological half-lives, serum protein binding, placental transfer, fetal accumulation, lactational transfer, and congener-specific elimination, are incorporated across the framework.

### 3.1 Placental development and function as an upstream target of PFAS toxicity

Beyond serving as a transfer interface, the placenta may represent an active target organ for PFAS developmental toxicity. PFAS have been detected in placental tissue and cord blood, confirming fetal exposure and indicating that these compounds can interact directly with the maternal–fetal interface (56, 57). Experimental studies using trophoblast cell models show that PFOS, PFOA, PFBS, and GenX can impair trophoblast migration and invasion, processes required for normal spiral artery remodeling and placental implantation (58). Disruption of these processes may contribute to placental insufficiency, a well-established pathway to fetal growth restriction.

PFAS may also affect placental vascular and endocrine functions. In placental trophoblast models, PFAS exposure has been associated with altered angiogenic factor production, stress-response signaling, oxidative stress, and inflammatory pathways (59). PFAS exposure has also been associated with altered placental endocrine markers, including human chorionic gonadotropin-related measures in early pregnancy (60). These placental mechanisms provide a plausible explanation for epidemiological associations between prenatal PFAS exposure and small-for-gestational-age birth or reduced birth weight (57, 61). Nevertheless, direct human evidence demonstrating placental mediation remains limited, and future studies should combine paired maternal, placental, and cord blood PFAS measurements with placental histopathology, vascular markers, endocrine biomarkers, and formal mediation analyses.

### 3.2 Molecular initiating events: PFAS-receptor interactions

For several studied PFAS congeners, particularly long-chain Perfluoroalkyl Acids (PFAAs) such as PFOA and PFOS, receptor interactions represent plausible molecular initiating events. In metabolic regulation, activation of peroxisome proliferator-activated receptor gamma (PPARγ) is considered a central mechanism of PFAS toxicity (15, 62). Long-chain PFAS, such as PFOA and PFOS, can act as PPARγ agonists, with binding affinity influenced by carbon chain length and terminal functional groups (63). Through receptor activation, PFAS can alter downstream gene expression, promote lipid accumulation and adipocyte differentiation, and impair insulin sensitivity, thereby contributing to metabolic dysfunction in offspring (64).

PFAS also disrupt endocrine signaling through interactions with thyroid and sex hormone pathways. PFAS show strong affinity for transthyretin (TTR), competing with thyroxine (T4) and potentially reducing thyroid hormone availability to the developing brain (65, 66). In addition, PFAS can modulate estrogen and androgen receptor signaling, for example by enhancing estrogen-responsive transcription or antagonizing androgen receptor activity (15, 67). Other nuclear receptors, including constitutive androstane receptor (CAR) and pregnane X receptor (PXR), may also be involved, linking PFAS exposure to xenobiotic metabolism and energy homeostasis (68, 69). Table 4 summarizes the interactions between PFAS and various receptors.

**Table 4:** Receptor interactions between prenatal pfas exposure and developmental toxicity.

Molecular target	Interaction type	Key affected pathways	Downstream cellular and health effects	Representative compounds studies	Reference
PPARα/γ	Agonist (especially long-chain PFAS: PFOS, PFOA, PFNA)	• **Adipogenesis:** Upregulation of adipogenic transcription factors (PPARγ, C/EBPα, SREBP1c) • **Lipid metabolism:** Enhanced fatty acid uptake, triglyceride synthesis, and lipoprotein lipase activity • **Glucose homeostasis:** Altered insulin sensitivity and glucose transporter expression • **Inflammatory modulation:** Suppression ofanti-inflammatory adipokines, elevation of pro-inflammatory cytokines	• **Steatosis:** Hepatic lipid accumulation and nonalcoholic fatty liver disease (NAFLD) • **Obesity:** Accelerated adipocyte differentiation, increased visceral adiposity • **Metabolic syndrome:** Dyslipidemia, insulin resistance, impaired glucose tolerance • **Cardiovascular risk:** Endothelial dysfunction, atherosclerotic plaque formation	PFOA, PFOS, PFNA, PFHxS	(15, 62, 63)
TTR	Competitive binder (high-affinity displacement of T4/T3)	• **Thyroid hormone distribution:** Disruption ofT4 transport across blood-brain barrier and placenta • **Thyroid hormone metabolism:** Altered deiodinase activity and peripheral conversion (T4^ T3) • **Hypothalamic-pituitary-thyroid axis:** Feedback dysregulation with altered TSH secretion • **Cerebral cortex development:** Reduced local T3 availability during critical neurodevelopmental windows	• **Neurodevelopmental deficits:** Reduced IQ, impaired language development, attention deficits • **Auditory processing abnormalities:** Hearing impairment, delayed auditory maturation • **Motor coordination deficits:** Fine and gross motor skill delays • **Behavioral alterations:** Increased hyperactivity, emotional dysregulation • **Structural brain changes:** Altered cortical thickness, white matter integrity disruption	PFOS, PFOA, PFHxS, PFNA	(65, 66)
ER	Modulator (e.g., PFOS acts as mixed agonist/antagonist; structure-dependent activity)	• **Estrogen signaling:** Disruption of ERE-mediated transcriptional responses • **Hypothalamic-pituitary-gonadal axis:** Altered GnRH pulsatility and gonadotropin secretion • **Mammary gland development:** Impaired branching morphogenesis and ductal elongation • **Uterine and vaginal development:** Disrupted epithelial proliferation and differentiation • **Bone metabolism:** Altered osteoblast/osteoclast balance and BMD accrual	• **Altered reproductive development:** - Females: Earlier menarche, accelerated pubertal progression, reduced fecundity - Mammary gland: Hypoplastic development, increased susceptibility to carcinogenesis • **Endocrine disorders:** Estrogen dominance symptoms, menstrual irregularities • **Skeletal effects:** Reduced peak bone mass, increased fracture risk • **Cancer susceptibility:** Enhanced estrogen-responsive tissue proliferation	PFOA, PFOS, selected alternatives	(15, 67)
AR	Antagonist (e.g., PFOA, PFOS, PFHxS demonstrate competitive inhibition)	• **Androgen signaling:** Blockade of DHT-mediated gene transcription • **Wolffian duct development:** Impaired differentiation into epididymis, vas deferens, seminal vesicles •External genitalia masculinization: Reduced 5α-reductase activity and androgen-dependent tissue growth •Spermatogenesis: Disruption of Sertoli cell function and germ cell maturation •Prostate development: Impaired glandular budding and branching	**• Male reproductive tract maldevelopment:** - Cryptorchidism (undescended testes) - Hypospadias (abnormal urethral opening) - Reduced anogenital distance (AGD) •Semen quality deterioration: Reduced sperm count, motility, and morphology •Testicular dysgenesis syndrome: Testicular cancer risk, Leydig cell dysfunction •Masculinization deficits: Reduced penile size, scrotal abnormalities •Adult reproductive impairment: Infertility, subfertility, testosterone deficiency	PFOA, PFOS, selected alternatives	(15, 67)
CAR/PXR	Activator (ligand-dependent nuclear translocation and heterodimerization with RXR)	•Xenobiotic metabolism: Induction of CYP450 enzymes (CYP3A4, CYP2B6, CYP2C9) •Phase II metabolism: Upregulation of UGTs, SULTs, and GSTs •Phase III transport: Enhanced expression of efflux transporters (P-gp, MRP2, BCRP) •Bile acid homeostasis: Altered synthesis and enterohepatic circulation •Steroid hormone metabolism: Accelerated clearance of endogenous hormones •Energy metabolism: Cross-talk with PPAR, LXR, and FXR pathways	•Disruption of metabolic homeostasis: - Altered drug and endobiotic clearance rates - Therapeutic efficacy reduction for co-administered medications •Endocrine disruption: Enhanced elimination of thyroid hormones, steroids, and vitamins •Hepatomegaly: Enzyme induction-associated liver enlargement •Cholestatic risk: Bile acid accumulation and hepatotoxicity •Tumor promotion: Potential contribution to hepatic carcinogenesis •Nutritional status: Fat-soluble vitamin deficiency (A, D, E, K)	Mostly PFOA/PFOS/PFHxS/ PFNA in cohort studies	(68, 69)

References listed in the table indicate representative primary studies, cohort studies, experimental studies, systematic reviews, or meta-analyses supporting each summarized statement. The table represents the authors' synthesis of the cited literature rather than new primary data. PFAS, per- and polyfluoroalkyl substances; PPAR, peroxisome proliferator-activated receptor; TTR, transthyretin; ER, estrogen receptor; AR, androgen receptor; CAR, constitutive androstane receptor; PXR, pregnane X receptor; PFOS, perfluorooctane sulfonic acid; PFOA, perfluorooctanoic acid; PFNA, perfluorononanoic acid; PFHxS, perfluorohexane sulfonic acid; T4, thyroxine; T3, triiodothyronine; TSH, thyroid-stimulating hormone; DHT, dihydrotestosterone; GnRH, gonadotropin-releasing hormone; ERE, estrogen response element; C/EBPα, CCAAT/enhancer-binding protein alpha; SREBP1c, sterol regulatory element-binding protein 1c; CYP, cytochrome P450; UGT, UDP-glucuronosyltransferase; SULT, sulfotransferase; GST, glutathione S-transferase; P-gp, P-glycoprotein; MRP2, multidrug resistance-associated protein 2; BCRP, breast cancer resistance protein; LXR, liver X receptor; FXR, farnesoid X receptor; RXR, retinoid X receptor; AGD, anogenital distance; BMD, bone mineral density; NAFLD, non-alcoholic fatty liver disease; IQ, intelligence quotient.

### 3.3 Cellular key events: oxidative stress, inflammation and apoptosis

Building on these molecular initiating events, PFAS exposure triggers a series of interconnected cellular key events. Mitochondrial dysfunction and oxidative stress are early manifestations of PFAS toxicity (70, 71). PFAS impair electron transport chain function, disrupt mitochondrial membrane potential, and increase reactive oxygen species (ROS) production, leading to oxidative damage to lipids, proteins, and DNA, as well as depletion of antioxidant defenses such as glutathione (72, 73). The developing nervous system appears particularly vulnerable to oxidative stress, which may contribute to PFAS-induced neurodevelopmental toxicity (74).

PFAS also activate inflammatory signaling pathways, especially NF-κB, through ROS accumulation and receptor-mediated mechanisms such as Toll-like receptor 4 (TLR4) signaling (75, 76). This promotes the expression of proinflammatory cytokines, including Tumor Necrosis Factor-alpha (TNF-α), interleukin-6 (IL-6), and IL-1β, thereby sustaining a proinflammatory cellular environment (77). In addition, PFAS can induce apoptosis, as shown by increased embryonic neural apoptosis in zebrafish and elevated caspase-3/7 activity in mammalian cells (78). However, direct evidence for PFAS-induced apoptosis in human developmental contexts remains limited.

### 3.4 Molecular and epigenetic mechanisms: developmental reprogramming

Epigenetic reprogramming is a fundamental mechanism linking early-life environmental exposure to long-term health outcomes and is mainly mediated by DNA methylation, histone modifications, and non-coding RNA regulation (79, 80). Disruption of these tightly regulated processes during critical developmental windows can induce persistent changes in gene expression, thereby affecting organ development and metabolic homeostasis and increasing disease susceptibility later in life.

#### 3.4.1 DNA methylation

At the DNA methylation level, birth cohort studies have demonstrated that prenatal PFAS exposure is associated with altered genome-wide DNA methylation patterns in neonatal cord blood (81–83). These changes may result from disruption of DNA methyltransferases (DNMTs) and ten-eleven translocation (TET) enzyme activity during critical developmental windows (54, 84). PFAS-associated methylation changes affect not only global patterns but also genes involved in metabolic regulation and neurodevelopment, potentially increasing susceptibility to obesity, type 2 diabetes, and dyslipidemia in offspring (85). Importantly, some of these methylation signatures persist from birth into adolescence, suggesting that DNA methylation may serve as a lasting molecular memory of early-life PFAS exposure (86).

#### 3.4.2 Histone modification

Prenatal PFAS exposure has also been associated with altered histone modifications. Studies report increased H3K4me3 and decreased H3K27me3 in children's peripheral blood leukocytes following prenatal exposure, suggesting changes in chromatin accessibility and gene expression (87). These alterations affect key genes in pathways such as insulin-like growth factor (IGF) signaling, which is involved in metabolic regulation and aging-related processes (88). PFAS-induced histone changes have also been implicated in carcinogenic processes. In human mammary epithelial cells, PFOS exposure reduces H3K9 acetylation, whereas PFOA alters H3K9me2 and H3K4me3 levels, changes that may promote chromatin remodeling and silencing of tumor suppressor genes (89).

#### 3.4.3 Non-coding RNA

Non-coding RNAs (ncRNAs), comprising microRNAs (miRNAs) and long non-coding RNAs (lncRNAs), are important regulators of gene expression and chromatin organization, and their dysregulation provides insight into the epigenetic toxicity of PFAS (90). PFAS exposure can alter ncRNA expression through epigenetic mechanisms; for example, hypermethylation of the lncRNA Maternally Expressed Gene 3 (MEG3) promoter suppresses MEG3 and its intron-derived miR-770, leading to dysregulated target gene expression and impaired placental cell growth (90). PFAS exposure has also been associated with altered methylation of multiple lncRNAs in mammary epithelial cells, suggesting potential effects on tumor suppressor function and malignant transformation (91). In addition, PFAS can suppress several miRNAs involved in tumorigenesis, cardiovascular regulation, and inflammation, including miR-101-3p, miR-144-3p, miR-19a-3p, miR-451, miR-23a, and miR-24 (92). Together, these multi-omics findings suggest several candidate mechanistic biomarkers for prenatal PFAS exposure, including PFAS-associated CpG methylation signatures in cord blood or placenta, ncRNAs such as MEG3/miR-770, miRNAs involved in inflammatory, cardiovascular, and metabolic regulation, and metabolomic features related to lipid metabolism, amino acid metabolism, mitochondrial stress, oxidative stress, and liver injury. However, these markers remain exploratory, and their sensitivity, specificity, and predictive value for offspring health outcomes have not been consistently validated across independent cohorts.

### 3.5 Gut microbiome alteration and the gut-brain-microbiome axis

Emerging evidence suggests that disruption of the gut microbiome may represent an additional pathway linking prenatal PFAS exposure to adverse developmental outcomes. The gut microbiota plays a critical role in immune maturation, metabolic homeostasis, and neurodevelopment through bidirectional communication along the gut–brain axis. Experimental studies have shown that PFAS exposure can alter gut microbial diversity and composition, accompanied by changes in microbial metabolites, intestinal barrier function, and inflammatory signaling. These alterations may influence host metabolism through modulation of lipid and glucose homeostasis and may affect neurodevelopment through immune activation, neuroinflammation, and altered production of microbiota-derived signaling molecules (5, 93–95).

Although several studies have reported associations between PFAS exposure and gut microbiome perturbations, direct evidence demonstrating that microbiome alterations mediate neurodevelopmental or metabolic outcomes in offspring remains limited. Most available data originate from animal models or cross-sectional investigations, and few studies have integrated prenatal PFAS exposure assessment, offspring microbiome profiling, and long-term health outcomes within the same cohort. Therefore, the gut–brain–microbiome axis should currently be regarded as a biologically plausible but incompletely established mechanism that warrants further investigation using longitudinal cohort studies, multi-omics approaches, and causal mediation analyses (95–98).

## 4 The evolving challenge: emerging PFAS alternatives and mixture effects

Although substantial attention has been given to legacy PFAS, the progressive phase-out of compounds such as PFOA and PFOS has introduced new challenges related to replacement chemicals and complex mixture exposures. This section summarizes the current evidence on emerging PFAS alternatives and real-world mixture scenarios.

### 4.1 Emerging PFAS alternatives: risk-benefit considerations and regrettable substitution

The increasing use of PFAS alternatives, including 6:2 Cl-PFESA, GenX, and 6:2 fluorotelomer sulfonic acid (6:2 FTSA), raises concerns about developmental toxicity. Biomonitoring studies have detected several of these alternatives in maternal blood, placental tissues, and umbilical cord blood, indicating that fetal exposure may occur during pregnancy (99–102). Table 5 outlines the toxicological characteristics of emerging alternatives. Experimental and epidemiological studies further suggest potential effects on thyroid hormone homeostasis, fetal growth, neurodevelopment, and metabolic regulation (100, 103–106). A balanced risk–benefit evaluation is therefore required for emerging PFAS alternatives. Their potential benefits include reducing reliance on legacy long-chain PFAS, lowering bioaccumulation potential for some short-chain compounds, and preserving critical technical functions in applications where non-fluorinated substitutes are not yet available. However, these benefits are use-specific and do not imply developmental safety. Many replacement PFAS remain highly persistent, more mobile in aquatic environments, difficult to remove, and insufficiently characterized with respect to placental transfer, fetal toxicokinetics, endocrine disruption, immunotoxicity, and developmental outcomes. To prevent regrettable substitution, replacement decisions should follow an essential-use and function-based alternatives assessment framework, prioritize non-fluorinated substitutes where feasible, require premarket developmental and mixture-toxicity testing, incorporate life-cycle and exposure assessment, and include post-market biomonitoring for pregnant women, infants, and other vulnerable populations (107, 108).

**Table 5:** Emerging PFAS alternatives: exposure and early toxicity evidence.

Alternative PFAS	Evidence summary	Developmental concern	References
6:2 Cl-PFESA (F-53B)	Detected in maternal blood, placenta, cord blood; thyroid/neurodevelopment concern	Placental transfer, endocrine/neurodevelopmental toxicity	(99–102)
GenX (HFPO-DA)	Detected in cord blood; shorter half-life but developmental toxicity signals	Placental dysfunction, fetal growth restriction	(100, 103–106)
6:2 FTSA	Detected in prenatal matrices; possible steroidogenic disruption	Fetal growth and endocrine effects	(102, 106)

References listed in the table indicate representative primary studies, cohort studies, experimental studies, systematic reviews, or meta-analyses supporting each summarized statement. The table represents the authors' synthesis of the cited literature rather than new primary data. 6:2 Cl-PFESA, 6:2 chlorinated polyfluoroalkyl ether sulfonate; GenX, hexafluoropropylene oxide dimer acid; HFPO-DA, Hexafluoropropylene Oxide Dimer Acid; 6:2 FTSA, 6:2 fluorotelomer sulfonic acid.

### 4.2 The complex reality of mixture exposure

In real-world scenarios, pregnant women and children are exposed to PFAS as mixtures rather than as isolated compounds. These mixtures may include both legacy and emerging PFAS, as well as other environmental contaminants such as heavy metals, plasticizers, and persistent organic pollutants. PFAS share several developmental toxicity mechanisms with other endocrine-disrupting chemicals (EDCs), including phthalates, bisphenol A (BPA), and polybrominated diphenyl ethers (PBDEs). Common pathways include thyroid hormone disruption, estrogen/androgen signaling interference, PPAR-mediated metabolic programming, oxidative stress, mitochondrial dysfunction, inflammation, epigenetic remodeling, and placental dysfunction (109, 110). However, PFAS also differ from many other EDCs in their high persistence, strong serum protein binding, compound-specific placental transfer, and long biological half-lives for several legacy congeners. Phthalates are generally shorter-lived compounds with prominent anti-androgenic and steroidogenic effects (111); BPA acts mainly through estrogen receptor-, G Protein-Coupled Estrogen Receptor (GPER)-, and metabolic signaling pathways (109, 110, 112); and PBDEs are lipophilic persistent pollutants that strongly disrupt thyroid hormone signaling and neurodevelopment (109, 113).

In mixtures, PFAS and other EDCs may interact through both toxicodynamic and toxicokinetic mechanisms. Additive or synergistic effects may occur when chemicals converge on shared pathways such as thyroid disruption, oxidative stress, placental dysfunction, or adipogenic/metabolic programming (13, 114, 115). Toxicokinetic interactions may arise through competition for serum proteins, placental transporters, or metabolic pathways, whereas antagonistic interactions are possible when chemicals exert opposing receptor-level effects. Therefore, co-exposure to PFAS, phthalates, BPA, PBDEs, and other contaminants may alter both the magnitude and direction of observed associations. Future studies should integrate mixture models with mechanistic biomarkers and multi-omics profiling to distinguish shared pathway effects from chemical-specific toxicity (113, 115).

Combined exposures may produce additive, synergistic, or antagonistic effects, complicating interpretation based on single-chemical toxicology. Mixture-based epidemiological approaches, including exposome frameworks, Bayesian kernel machine regression, and weighted quantile sum regression, have begun to capture these combined effects and have identified associations with growth, metabolic, and immunological outcomes (116–120). Methodological advances are also needed to better characterize PFAS mixtures. Weighted Quantile Sum (WQS) regression is useful for estimating the overall effect of correlated mixtures and identifying influential components when effects are assumed to act in the same direction. Quantile g-computation provides greater flexibility by accommodating both positive and negative component effects. Bayesian Kernel Machine Regression (BKMR) is particularly valuable for evaluating non-linear exposure–response relationships and potential interactions among PFAS congeners. Because PFAS exposures are often highly correlated, mixture-oriented methods are generally preferred over conventional multi-pollutant regression models. Future studies should compare findings across multiple analytical approaches and explicitly evaluate potential interactions among legacy PFAS, emerging alternatives, and co-occurring environmental contaminants.

.

## 5 Evidence synthesis and critical appraisal: gaps, uncertainties and future research directions

Recent research has highlighted the health impacts of prenatal PFAS exposure, particularly on offspring, including epidemiological findings, toxicological mechanisms, and challenges posed by substitutes and mixture exposures. However, uncertainties remain about the confidence in these associations, requiring systematic evaluation to identify critical gaps and guide future research.

### 5.1 Strength of the evidence and causality

Building on the descriptive epidemiological overview presented in Section 2, this section focuses on the strength, consistency, and causal interpretation of the evidence. Among the outcomes reviewed, the most robust evidence supports associations between prenatal PFAS exposure and impaired immune function, particularly reduced vaccine antibody responses, as well as altered fetal growth and postnatal metabolic trajectories. These conclusions are supported by relatively consistent findings from prospective birth cohorts and meta-analyses. In contrast, evidence for neurodevelopmental and endocrine outcomes remains suggestive but less consistent. Reported associations with cognitive function, behavioral outcomes, thyroid hormones, and reproductive development vary across studies, likely reflecting differences in exposure windows, PFAS congeners, outcome definitions, child age at assessment, and adjustment for confounders. Evidence for hepatic, renal, cardiovascular, and cellular aging endpoints is still emerging and should be interpreted cautiously because of fewer studies and limited replication.

Causal inference remains constrained by several methodological limitations, including exposure misclassification from single-time-point PFAS measurements, co-exposure to other environmental contaminants, residual confounding, sex-specific and congener-specific differences, and limited long-term follow-up. Therefore, while the epidemiological evidence indicates biologically plausible associations between prenatal PFAS exposure and multiple offspring outcomes, the level of confidence differs substantially across endpoints. Future studies should prioritize repeated exposure assessment, standardized outcome measurement, mixture modeling, and integration of mechanistic biomarkers to strengthen causal interpretation.

Using the Bradford Hill-informed framework, immune and growth/metabolic outcomes showed the strongest causal support, based on temporality, relatively consistent cohort findings, biological plausibility, and coherence with mechanistic evidence. In contrast, neurodevelopmental, endocrine, hepatic, renal, cardiovascular, and cellular aging endpoints met fewer Bradford Hill considerations because of inconsistent findings, heterogeneous exposure and outcome assessment, limited replication, and potential residual confounding. These outcomes were therefore classified as suggestive or emerging rather than conclusive.

Sex-specific effects were also considered in evidence interpretation. The clearest sex-related patterns were observed for growth/metabolic and endocrine/reproductive outcomes. Several studies reported stronger associations between prenatal PFAS exposure and postnatal BMI gain, adiposity, or obesity risk in girls, whereas endocrine and reproductive endpoints showed sex-dependent associations such as altered pubertal timing in girls, delayed genital development in boys, and changes in cord blood sex hormone levels. Some studies also suggested stronger associations with ADHD-related outcomes or blood pressure in boys, and allergic outcomes in girls, but these findings were less consistently replicated. Potential mechanisms include sex-dependent placental transfer and fetal accumulation, differences in hormonal milieu, disruption of estrogen/androgen and thyroid signaling, PPAR-mediated metabolic programming, immune maturation, mitochondrial stress, and epigenetic regulation. Therefore, sex-specific findings should be interpreted cautiously but incorporated into future causal appraisal and developmental risk assessment.

Adjustment for confounding varied across epidemiological studies. Most prospective birth cohorts adjusted for core maternal and sociodemographic factors, including maternal age, parity, pre-pregnancy BMI, smoking status, education, or socioeconomic status. However, adjustment for diet-related exposure sources, particularly fish and seafood intake, breastfeeding duration, and co-exposures to other persistent organic pollutants, was less consistent across studies (13, 28, 37, 120). This inconsistency may contribute to residual confounding, because maternal diet, breastfeeding practices, socioeconomic conditions, and co-occurring contaminants can be associated with both PFAS exposure levels and offspring health outcomes. For example, fish intake may increase PFAS exposure while also providing beneficial nutrients, whereas breastfeeding may both increase postnatal PFAS exposure and influence immune and growth outcomes. Therefore, residual confounding could either exaggerate or attenuate observed associations, depending on the direction of these relationships. Future studies should apply harmonized covariate adjustment, repeated exposure assessment, negative-control or sensitivity analyses, and mixture-based models to better separate PFAS-specific effects from correlated lifestyle, nutritional, and environmental factors.

For major outcomes, reported effect sizes are generally modest but potentially important at the population level (28, 37). Meta-analyses and large birth cohorts have reported reductions in birth weight of approximately 50–100 g associated with higher prenatal PFAS exposure, while prospective cohorts have observed roughly 10%−20% higher risks of childhood overweight or obesity. For immune outcomes, systematic reviews have reported approximately 10%−30% lower vaccine antibody concentrations among children with higher prenatal or early-life PFAS exposure. Although these effect sizes are relatively small for individual children, the near-ubiquitous nature of PFAS exposure means that even modest shifts in growth, metabolic, or immune outcomes may translate into substantial public health burdens.

Dose–response relationships appear to differ across outcome domains. For immune outcomes, particularly vaccine antibody responses, available evidence is most consistent with a monotonic inverse association, and a clear safe threshold has not been consistently identified. For growth and metabolic outcomes, dose–response patterns appear more complex and may vary by developmental stage, sex, PFAS congener, and outcome timing. Endocrine-related outcomes may plausibly show non-monotonic responses because PFAS can interfere with hormone signaling pathways, although epidemiological confirmation remains limited. For neurodevelopmental, hepatic, renal, cardiovascular, and cellular aging endpoints, current evidence remains insufficient to determine whether the dose–response pattern is linear, threshold-based, or non-monotonic.

### 5.2 Knowledge gaps and methodological limitations

Several cross-cutting knowledge gaps limit interpretation of the evidence summarized above. I. toxicokinetic information remains incomplete for many emerging PFAS and replacement compounds, particularly with respect to pregnancy-specific distribution, placental transfer efficiency, fetal accumulation, and postnatal elimination. PBPK/PBTK-based fetal exposure estimation requires compound- and pregnancy-specific data on maternal kinetics, protein binding, clearance, placental transfer, fetal distribution/elimination, and lactational transfer. For emerging PFAS alternatives such as 6:2 Cl-PFESA, GenX/Hexafluoropropylene Oxide Dimer Acid (HFPO-DA), and 6:2 FTSA, key data on pregnancy-specific half-lives, placental transfer, fetal kinetics, and breast milk partitioning remain largely unavailable. II. long-term follow-up data are limited, making it difficult to determine whether early biological changes translate into persistent metabolic, immune, neurodevelopmental, or endocrine disorders later in life. III. comparison across studies is hindered by heterogeneity in exposure assessment, including differences in biological matrices, sampling time points, PFAS panels, and treatment of values below detection limits. Outcome definitions and assessment ages also vary substantially, reducing comparability across cohorts. A further limitation is the reliance on single-time-point PFAS measurements during pregnancy. Although several legacy PFAS have long biological half-lives, one measurement may not capture gestational variation or trimester-specific susceptibility windows. Pregnancy-related changes in plasma volume, renal clearance, protein binding, parity, breastfeeding history, and placental transfer may alter measured PFAS concentrations and introduce exposure misclassification, particularly for fetal growth outcomes (121, 122). Matrix differences also reduce cross-study comparability. Maternal serum or plasma reflects maternal circulating burden at sampling, whereas cord serum or plasma more closely approximates fetal exposure near delivery but is influenced by placental transfer efficiency, gestational age, fetal–placental kinetics, protein binding, transporter activity, and PFAS chain length or functional group. Therefore, maternal and cord blood concentrations should not be treated as interchangeable exposure metrics. Future studies should use repeated and paired maternal–placental–cord sampling, harmonized analytical methods, and PBPK/PBTK modeling to improve fetal internal dose estimation (123, 124). IV. causal inference is complicated by co-exposure to other environmental contaminants, residual confounding, sex-specific susceptibility, and congener-specific toxicokinetics. Finally, mechanistic and epidemiological studies remain insufficiently integrated. Animal and *in vitro* models provide important biological insights, but interspecies differences and exposure-level discrepancies limit direct extrapolation to human pregnancy. Bridging these gaps will require repeated exposure measurements, standardized outcome assessment, mixture-oriented statistical models, human-relevant experimental systems, and integration of mechanistic biomarkers into prospective birth cohorts.

### 5.3 Pioneering future research

To address knowledge gaps, innovative methodologies are needed. Human-relevant models, like pluripotent stem cell-derived organoids, can help study the developmental toxicity of PFAS and mechanisms in key organs. Computational toxicology methods, including molecular simulations, could identify potential PFAS compounds and mechanisms. Expanding large birth cohorts with repeated exposure assessment and multi-omics profiling may help identify and validate candidate biomarkers, including CpG methylation signatures, ncRNAs/miRNAs, and metabolites related to lipid metabolism, mitochondrial stress, oxidative stress, and liver injury. At present, these biomarkers should be regarded as exploratory or mechanistic rather than clinically validated predictors, because their sensitivity, specificity, and positive or negative predictive values have not been consistently established. Future studies should test their reproducibility across independent cohorts and evaluate whether they improve prediction of immune, metabolic, neurodevelopmental, or endocrine outcomes beyond exposure measurements and conventional clinical risk factors. Additionally, PBPK/PBTK models could refine risk assessment for emerging PFAS substitutes by linking maternal biomonitoring data with fetal internal dose estimates. Future model development should prioritize paired maternal blood, placenta, cord blood, meconium, and breast milk measurements, together with *in vitro* placental transport data and human-relevant kinetic parameters, to reduce uncertainty in fetal exposure estimation. In this context, PBPK/PBTK models should not be viewed as standalone risk-assessment tools, but as quantitative modules linking external exposure, maternal serum concentrations, placental transfer, fetal internal dose, and postnatal exposure. Required inputs include congener-specific half-lives, serum protein binding, gestational physiology, placental transfer parameters, breastfeeding-related exposure, and longitudinal biomonitoring data. For emerging PFAS substitutes, these parameters are often unavailable; therefore, model development should proceed in parallel with targeted toxicokinetic and biomonitoring studies.

## 6 Implications for intergenerational hazard assessment and risk management

Current PFAS risk assessment methods, which focus on individual chemicals and adult animal data, fail to capture the persistent, intergenerational health impacts of PFAS exposure during critical developmental windows. A new assessment framework is needed to reflect real-world exposure and biological complexity.

### 6.1 Rethinking risk assessment paradigms

Evidence shows increased susceptibility to pollutants during early life stages (125). Developing organisms, unlike adults, undergo critical physiological changes (126), and prenatal PFAS exposure may lead to health risks in future generations. Nevertheless, current assessment frameworks largely neglect this critical dimension. Integrating developmental and intergenerational effects into risk assessment is essential, requiring reforms such as improved developmental toxicity testing, multigenerational animal studies where appropriate, careful evaluation of true transgenerational designs, and the use of validated epigenetic biomarkers. Risk assessment should also account for potential sex-specific susceptibility. Future studies and regulatory evaluations should require sex-stratified reporting, formal exposure-by-sex interaction testing, and sex-specific biomarkers or endpoints where biologically justified. When consistent evidence indicates greater vulnerability in one sex, developmental uncertainty factors or risk characterization should consider this differential susceptibility rather than assuming uniform effects across male and female offspring.

To avoid terminological ambiguity, intergenerational and transgenerational effects should be distinguished. In the context of maternal gestational exposure, intergenerational effects refer to outcomes in directly exposed generations, including the F1 fetus and potentially the F2 germline developing within the F1 fetus. True transgenerational effects require persistence of exposure-associated phenotypes or molecular alterations in an unexposed generation; for maternal gestational exposure, this is generally the F3 generation (127). Accordingly, the present review uses “intergenerational” to describe the available human evidence on prenatal PFAS exposure and offspring outcomes (128). Although experimental studies suggest that PFAS may induce multigenerational or transgenerational changes in some models, robust human evidence for true PFAS-associated F3 transgenerational effects is currently lacking (129).

### 6.2 Informing public health policy and regulatory action

Public health policies and regulations should address PFAS risk more effectively. Stricter drinking water standards for legacy PFAS, monitoring for emerging substitutes, and food safety regulations targeting PFAS-prone food categories are necessary. Risk management should prioritize compounds based on their toxicity, persistence, and bioaccumulation. Special protection should be provided to vulnerable groups, particularly pregnant women and infants, with safety margins for developmental exposures. These uncertainties have important implications for health-based guidance values. Because a clear threshold has not been established for several developmental endpoints, guidance values should not rely solely on an assumed threshold model derived from adult toxicity data. Instead, risk assessment should incorporate flexible dose–response modeling, sensitive developmental windows, PFAS-specific toxicokinetics, mixture exposure, and additional uncertainty factors for prenatal and early-life susceptibility. For endocrine-related endpoints, the possibility of non-monotonic responses further supports a precautionary approach and the use of low-dose data when deriving protective guidance values.

### 6.3 Communicating risk and protecting vulnerable populations

Risk communication should be based on biomonitoring data and include tiered interventions. At the individual level, PFAS screening for high-risk groups and guidance on water treatment and diet are essential. At the community level, interventions should include alternative drinking water and risk communication through healthcare providers. Future strategies must be culturally tailored and rigorously evaluated for effectiveness to protect vulnerable populations.

### 6.4 A Developmental risk assessment framework for prenatal PFAS exposure

To align the framework with the internationally recognized risk assessment paradigm, Figure 3 follows the standard four-step structure of hazard identification, hazard characterization, exposure assessment, and risk characterization. Developmental and PFAS-specific considerations are incorporated within each step, rather than being presented as separate or parallel components.

To operationalize the internationally recognized four-step risk assessment framework for prenatal PFAS exposure, we adapt it to explicitly reflect developmental and PFAS-specific considerations. Hazard identification focuses on evidence linking specific PFAS congeners or mixtures to adverse developmental outcomes, integrating epidemiological, experimental, and mechanistic findings with emphasis on sensitive developmental windows and key offspring endpoints including immune, metabolic, neurodevelopmental, and endocrine effects. Hazard characterization evaluates dose–response relationships while explicitly accounting for PFAS-specific toxicokinetics, including long biological half-lives, serum protein binding, placental transfer, fetal accumulation, and developmental-stage-specific elimination, alongside uncertainty in deriving health-based guidance values. Exposure assessment integrates external and internal exposure information, including dietary and drinking-water intake as well as biomonitoring data from maternal serum, placenta, cord blood, breast milk, and infant matrices, with particular attention to mixture exposures and temporal variability during pregnancy and early life. Risk characterization then synthesizes hazard and exposure information to estimate population-level risk, incorporating margins of exposure, susceptible developmental windows, sex-specific vulnerability, and mixture-related uncertainty. PBPK/PBTK modeling, multi-omics biomarkers, and experimental systems are positioned as complementary tools to refine internal dose estimation and mechanistic interpretation within this framework.

### 6.5 Complementing existing regulatory assessments through a developmental lens

Existing agency assessments provide essential foundations for PFAS regulation, including health-based guidance values, chemical-specific toxicity values, and carcinogenic hazard classifications. However, regulatory assessments are usually designed for specific decision contexts, such as food safety, drinking-water regulation, or cancer hazard classification. As a result, they may not fully integrate prenatal exposure timing, placental transfer, fetal internal dose, offspring health trajectories, developmental mechanisms, and intergenerational susceptibility.

The contribution of the present review is therefore complementary. First, it centers the prenatal and early-life window as the primary unit of interpretation rather than treating pregnancy as only one subgroup within a broader population assessment. Second, it organizes offspring outcomes across immune, growth/metabolic, neurodevelopmental, endocrine, hepatic, renal, and cardiovascular domains, while explicitly distinguishing stronger evidence from suggestive or emerging evidence. Third, it links epidemiological findings with developmental mechanisms, including placental dysfunction, thyroid hormone transport disruption, receptor-mediated signaling, mitochondrial stress, immune modulation, microbiome alteration, and epigenetic reprogramming. Fourth, it highlights evidence gaps for emerging PFAS alternatives and real-world mixtures, which remain incompletely covered in most regulatory assessments. Finally, it outlines how repeated biomonitoring, human-relevant experimental systems, multi-omics biomarkers, mixture modeling, and PBPK/PBTK approaches could be integrated into future developmental hazard assessment.

Thus, this review should not be interpreted as a competing regulatory assessment. Instead, it provides a developmental and intergenerational perspective that may help identify research priorities, refine causal interpretation, and support more protective evaluation of prenatal and early-life PFAS exposure.

## 7 Conclusion

Prenatal exposure to several measured PFAS, particularly legacy PFAAs such as PFOA, PFOS, PFHxS, and PFNA, is a developmental health concern, but the strength and generalizability of evidence differ substantially across congeners and outcome domains. The strongest human evidence is currently derived from studies of legacy PFAS, whereas data for short-chain PFAS, ether-based alternatives, fluorotelomer compounds, and other emerging substitutes remain limited. The most consistent human evidence supports impaired vaccine antibody responses and altered growth or metabolic trajectories, while neurodevelopmental, endocrine, hepatic, renal, cardiovascular, and cellular aging endpoints remain suggestive or emerging. Mechanistic evidence indicates that placental transfer, receptor-mediated signaling, thyroid hormone transport disruption, mitochondrial and inflammatory stress, immune modulation, and epigenetic programming may contribute to offspring susceptibility. However, causal interpretation is limited by heterogeneous exposure assessment, outcome definitions, co-exposures, congener-specific kinetics, and limited long-term follow-up. Compared with existing agency assessments that primarily address selected PFAS in regulatory or carcinogenicity contexts, this review provides a complementary developmental-health synthesis focused on prenatal exposure, offspring outcomes, placental and fetal mechanisms, emerging alternatives, mixture exposure, and intergenerational risk assessment. The strongest evidence currently supports immune and growth/metabolic outcomes, whereas other endpoints and emerging alternatives require further validation. Future work should operationalize this developmental framework through repeated biomonitoring, congener-specific toxicokinetic data, human-relevant models, multi-omics biomarkers, mixture methods, and PBPK/PBTK modeling.
